# Biochemical and Immunological Characterization of *Toxoplasma gondii* Macrophage Migration Inhibitory Factor*

**DOI:** 10.1074/jbc.M112.419911

**Published:** 2013-02-26

**Authors:** Caroline Sommerville, Julia M. Richardson, Roderick A. M. Williams, Jeremy C. Mottram, Craig W. Roberts, James Alexander, Fiona L. Henriquez

**Affiliations:** From the ‡ Strathclyde Institute of Pharmacy and Biomedical Sciences, University of Strathclyde, Glasgow G4 0RE, Scotland, United Kingdom,; § School of Biological Sciences, University of Edinburgh, Mayfield Road, Edinburgh EH9 3JR, Scotland, United Kingdom,; ‖ Wellcome Trust Centre for Molecular Parasitology, Institute of Infection, Immunity and Inflammation, College of Medical, Veterinary and Life Sciences, University of Glasgow, Glasgow G12 8TA, Scotland, United Kingdom, and; ¶ Institute of Biomedical and Environmental Health Research School of Science, University of the West of Scotland, Paisley PA1 2BE, Scotland, United Kingdom

**Keywords:** Cell Signaling, Cellular Immune Response, Crystallography, Enzyme Kinetics, Macrophages, Parasite, Toxoplasma, Biochemistry, Immunology, Mammalian Macrophage Migration Inhibitory Factor

## Abstract

Macrophage migration inhibitory factor (MIF) is a proinflammatory molecule in mammals that, unusually for a cytokine, exhibits tautomerase and oxidoreductase enzymatic activities. Homologues of this well conserved protein are found within diverse phyla including a number of parasitic organisms. Herein, we produced recombinant histidine-tagged *Toxoplasma gondii* MIF (TgMIF), a 12-kDa protein that lacks oxidoreductase activity but exhibits tautomerase activity with a specific activity of 19.3 μmol/min/mg that cannot be inhibited by the human MIF inhibitor ISO-1. The crystal structure of the TgMIF homotrimer has been determined to 1.82 Å, and although it has close structural homology with mammalian MIFs, it has critical differences in the tautomerase active site that account for the different inhibitor sensitivity. We also demonstrate that TgMIF can elicit IL-8 production from human peripheral blood mononuclear cells while also activating ERK MAPK pathways in murine bone marrow-derived macrophages. TgMIF may therefore play an immunomodulatory role during *T. gondii* infection in mammals.

## Teaser

**Background:** *Toxoplasma gondii* possesses proteins that can modulate host immune responses. TgMIF is a homologue of mammalian macrophage migration inhibitory factor.

**Results:** TgMIF possesses tautomerase but not oxidoreductase activity. It can stimulate ERK MAPK and IL-8 production.

**Conclusion:** TgMIF is capable of modulating host molecules.

**Significance:** The study identifies immunomodulatory activity of TgMIF consistent with a role for sustaining parasite survival.

## Introduction

*Toxoplasma gondii* is an obligate intracellular protozoan parasite of significant economical and public health importance. Infection with *T. gondii* is common, and it is estimated that ∼30% of the world's population is chronically infected with this parasite. Typically, infection with *T. gondii* results in the induction of a classical type 1 response with the production of proinflammatory cytokines such as IFN-γ by natural killer cells and T cells controlling acute infection characterized by the rapidly dividing tachyzoite (1, 2). This response is not only essential for host survival, but it also creates an environment that is favorable for maintaining parasite survival via the establishment of a chronic latent infection characterized by a stage switch from tachyzoite to slow dividing bradyzoite (3). Overproduction of proinflammatory mediators can result in severe pathology; and therefore, the induction of regulatory mediators such as IL-10 is necessary to limit immune pathology during the course of infection (4). The parasite plays a significant direct role in influencing disease outcome, and recent studies have demonstrated the ability of *T. gondii* to modulate host immune responses via the activity of a number of parasite-specific proteins including cyclophilin-18 and heat shock protein 70 (for reviews, see Refs. 5–7).

MIF^2^ is an ancient proinflammatory mammalian cytokine that has been shown to participate in both innate and adaptive immune responses (for reviews, see Refs. 8–10). It is highly conserved in mammalian species with 86% amino acid sequence identity among MIF in cat, mouse, and humans. The proinflammatory activity of MIF has been implicated in a diverse range of biological processes including activation of ERK MAPK pathways in fibroblasts (11) and inhibition of the anti-inflammatory actions of glucocorticoids (12). MIF has also been shown to induce production of proinflammatory mediators such as TNF-α, IL-8, and IL-17 (12–14) while maintaining macrophage proinflammatory function by inhibiting p53-dependent apoptosis of macrophages (15). Despite a clear role for MIF as a proinflammatory mediator, it has also been suggested that MIF may mediate anti-inflammatory activities via binding Jab-1 and preventing AP-1 proinflammatory gene expression (16). Significantly, homologues of MIF have been described in many parasitic species including *Plasmodium*, *Leishmania*, *Brugia*, *Clostridium*, and *Eimeria*, and it has been implied that these proteins facilitate the manipulation of the host immune response during infection (17–21).

Herein, we report the biochemical and immunological characterization of a homologue of MIF in *T. gondii*, TgMIF, for which the crystal structure has been solved to 1.82-Å resolution. Furthermore, we have shown that TgMIF is a biochemically active protein that is capable of inducing IL-8 production from human peripheral blood mononuclear cells (PBMCs) while also activating ERK MAPK pathways in murine bone marrow-derived macrophages. The existence of TgMIF may facilitate parasitism in the definitive (Felidae), intermediate (*Mus musculus*), and incidental (*Homo sapiens*) hosts.

## EXPERIMENTAL PROCEDURES

### 

#### 

##### Alignments

**FIGURE 1.**
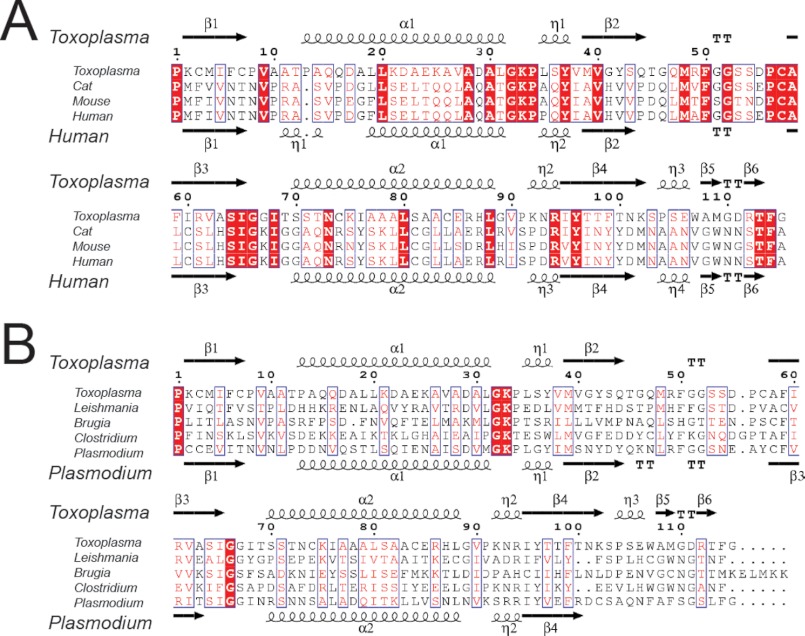
**Amino acid alignment of MIF sequences from *T. gondii* (ACY01255) and from host species (domestic cat (NC_018734. 1), human (NP_002406), and mouse (NP_034928)) (*A*) and other pathogens (*L. major* (Q4Q413), *B. malayi* (AY004865), *Clostridium saccharolyticum* (CBK76204), and *Plasmodium falciparum* (XP_001350690)) (*B*).** Conserved residues are shown in *white* on a *red background,* and similar residues are highlighted in *red*. Secondary structure elements for TgMIF are indicated *above* the alignments and for human and *Plasmodium* MIF are indicated *below* the alignments in *A* and *B*, respectively. *TT* represents a β-turn.

MIF sequences were retrieved from NCBI. Sequence alignments were performed using Tcoffee, and ESpript was then used to assemble Fig. 1.

##### Cloning, Expression, and Purification of C-terminal His-tagged TgMIF

TgMIF was amplified from cDNA of the RH strain using primers that had been specifically designed to include NdeI and XhoI restriction sites (underlined) in the forward and reverse primers, respectively, for cloning into vector pET21a+. Primer sequences were as follows: forward primer, 5′-GGGGCATATGCCCAAGTGCATGATCTTTTGCC-3′; and reverse primer, 5′-GGGGCTCGAGGCCGAAAGTTCGGTCGCCCATGGC-3′. The reverse primer was designed to omit the stop codon and allow translation of the C-terminal His tag on the pET21a+ vector. The TgMIF ORF was amplified from *T. gondii* (RH strain) cDNA using a *Taq* and *Tgo* DNA polymerase as part of a high fidelity PCR system (Roche Applied Science). The 351-bp TgMIF ORF DNA product was subsequently ligated into expression vector pET21a+ (Novagen). *Escherichia coli* BL21 competent cells (Rosetta strain) were transformed, and expression was induced by the addition of 1 mm isopropyl 1-thio-β-galactopyranoside at 30 °C overnight. Cells were then lysed by sonication and the addition of lysozyme (1 μg/ml). Recombinant proteins were purified using Ni^2+^-nitrilotriacetic acid columns (Qiagen), eluted with 1 m imidazole, and stored in a Tris-HCl buffer, pH 8 at ∼6 mg/ml at −80 °C. For biological assays, endotoxin was removed from protein samples using the ProteoSpin Endotoxin Removal Maxi kit (Novagen), and endotoxin levels were then quantified using the QCL-1000 chromogenic *Limulus* amebocyte lysate end point assay (Cambrex).

##### Crystallization

Crystals were grown by the hanging drop, vapor diffusion method in 24-well Linbro plates. Crystals of TgMIF grew at 277 K over a well solution containing 1.82 m ammonium sulfate and 100 mm Tris, pH 6.5. The drop contained 2 μl of protein solution at 15 mg/ml in 50 mm Tris, pH 7.5 plus 2 μl of well solution. Prior to data collection, crystals were briefly immersed in a cryoprotectant solution containing 20% (v/v) glycerol plus well solution and then flash frozen in liquid nitrogen.

##### X-ray Data Collection and Structure Determination

All diffraction data were collected at 100 K on beam-line I03 at the Diamond Light Source (Oxfordshire, UK), indexed with iMosflm, and merged and scaled with SCALA (22). For molecular replacement, a homology model of the TgMIF structure was generated using the SwissModel server and used as the search model in PHASER (22). Structure building and refinement was performed using Coot (23) and Refmac, respectively. Data collection and refinement statistics are shown in Table 1. The structure has been deposited in the Research Collaboratory for Structural Bioinformatics Protein Data Bank under accession code 4DH4.

##### Enzyme Assays

Tautomerase assays were performed as described previously (24). Tautomerase activity was measured at 37 °C by the addition of 48 μl of l-DOPA methyl ester (10 mm) (ϵ = 3700 m^−1^ cm^−1^) to 32 μl of sodium periodate (20 mm) to which an equal volume of sodium phosphate buffer (10 mm, pH 6.2) with 1 mm EDTA was added. To start the reaction, 80 μg of TgMIF and 0.5 μg of human MIF (HsMIF) (R&D Systems) were added, and tautomerization of l-dopachrome methyl ester was then measured at λ_474 nm_. The inhibitory effect of (*S*,*R*)-3-(4-hydroxyphenyl)-4,5-dihydro-5-isoxazole acetic acid (ISO-1) (Sigma) was determined by the preincubation of 80 μg of TgMIF and 0.5 μg of HsMIF with 10 μm ISO-1 for 30 min prior to adding it to the substrate.

##### Culture of Human Peripheral Blood Mononuclear Cells

Human PBMCs were isolated from freshly drawn blood using Histopaque (Sigma-Aldrich). Isolated mononuclear cells were seeded to 96-well plates at 5 × 10^6^ cells/ml in RPMI 1640 medium containing 10% heat-inactivated fetal calf serum, 2 mm l-glutamine, 100 units/ml penicillin, and 100 μg/ml streptomycin and incubated at 37 °C in 5% CO_2_. Cells were stimulated with increasing concentrations of endotoxin-free TgMIF (1–1000 ng/ml) for 24 h following which cell supernatants were collected for analysis by ELISA.

##### IL-8 Assay

To detect IL-8 in cell supernatants, 96-well plates were coated with purified mouse anti-human IL-8 (Pharmingen) at 2 μg/ml in PBS, pH 9 and incubated overnight at 4 °C. Following the addition of supernatants to wells, recombinant human IL-8 was also added to corresponding wells in doubling dilutions ranging from 20 ng/ml to 387.5 pg/ml. Biotinylated anti-human IL-8 (Pharmingen) was added to each well at 1 μg/ml before the addition of streptavidin-alkaline phosphate (Pharmingen) diluted 1:2000. Finally, *p-*nitrophenyl phosphate (Sigma) at 1 mg/ml in glycine buffer was added, and absorbances were recorded at 450 nm. All assays were carried out in triplicate.

##### Isolation of Macrophages from Bone Marrow Stem Cells and Sample Preparation

Femurs were removed from male BALB/c mice and flushed using a 25-gauge needle with medium consisting of 30% L cell-conditioned supernatant, 20% heat-inactivated fetal calf serum, 2 mm l-glutamine, 100 units/ml penicillin, and 100 μg/ml streptomycin in Dulbecco's modified Eagle's medium supplemented with sodium pyruvate, glucose, and pyridoxine hydrochloride (Invitrogen). Flushed bone marrow stem cells were passed through a 21-gauge needle before being split between Petri dishes and incubated at 37 °C in 5% CO_2._ Cultures were then supplemented with media on days 3 and 7. Confluent macrophages were harvested and washed on day 10 and seeded at 1 × 10^6^/ml in RPMI 1640 medium supplemented with 10% heat-inactivated fetal calf serum, 2 mm l-glutamine, 100 units/ml penicillin, and 100 μg/ml streptomycin. Macrophages were incubated at 37 °C in 5% CO_2_ throughout the duration of the experiment.

Cells were stimulated with concentrations of endotoxin-free TgMIF ranging from 1 to 1000 ng/ml over a 24-h period. Control cells were stimulated with 1 ng/ml HsMIF for 24 h before samples were collected. To collect samples, cells were washed in PBS and harvested by agitation in 150 μl of sample buffer after which samples were boiled to denature proteins.

##### ERK MAPK Western Blot

Stimulated macrophage samples were resolved by 14% SDS-PAGE. Proteins were first resolved in a polyacrylamide gel and then transferred to a nitrocellulose membrane (Amersham Biosciences) using the Novex Xcell Blot Module (Invitrogen) at 30 V for 90 min. The membrane was blocked for 2 h with a blocking buffer containing 10% FCS in PBS after which the membrane was incubated overnight at 4 °C in the blocking buffer containing the mouse anti-ERK antibody (1:7500 dilution) (Santa Cruz Biotechnology). The membrane was washed for 90 min, changing the wash buffer (PBS containing 0.05% Tween 20) every 15 min. Anti-mouse HRP-linked secondary antibody (diluted 1:7500) (Santa Cruz Biotechnology) in blocking buffer was incubated with the membrane for 2 h at room temperature. Antibody binding was measured by chemiluminescence and incubation of 2 ml of ECL substrate (Pierce) for 2 min. The film was then developed in the XOMAT (Konica) developer.

## RESULTS

### 

#### 

##### Cloning and Expression of T. gondii MIF

*T. gondii* encodes a single copy of MIF that is expressed in both tachyzoite and bradyzoite forms of the parasite (data not shown). Alignment of TgMIF from all three strains shows 99.7% nucleotide sequence identity and 100% identity of amino acids (data not shown). Interspecies sequence alignments reveal that TgMIF has 59% amino acid sequence similarity and 26% identity with mammalian MIFs from host species (Fig. 1*A*) and 41.7% similarity with other parasite MIFs (Fig. 1*B*). Amino acid numbering in this study begins with Pro^1^ because the starting methionine is cleaved. The alignments highlight the conservation of the N-terminal Pro^1^, which mediates tautomerase activity, and additional conserved residues located around the active site, notably Lys^33^, Tyr^96^, and Ile^65^. This suggests that TgMIF should have tautomerase activity. In contrast, the C*XX*C motif in human MIF that is required for oxidoreductase activity is absent in TgMIF in that the Cys^59^ found in humans is Ile^60^ in TgMIF. In accordance with other MIF proteins, no classical secretory signal sequence has been identified in TgMIF (25).

Successful cloning of TgMIF ORF into the expression vector pET21a+ with a C-terminal His_6_ tag allowed recombinant expression of the protein in *E. coli* and subsequent purification using Ni^2+^-nitrilotriacetic acid columns (Qiagen). Approximately 12 mg of protein was purified per 1 liter of culture. The protein was used for structural analysis, biochemical analysis of N-terminal tautomerase function, and analysis of immunological activities.

##### Crystal Structure of TgMIF

**FIGURE 2.**
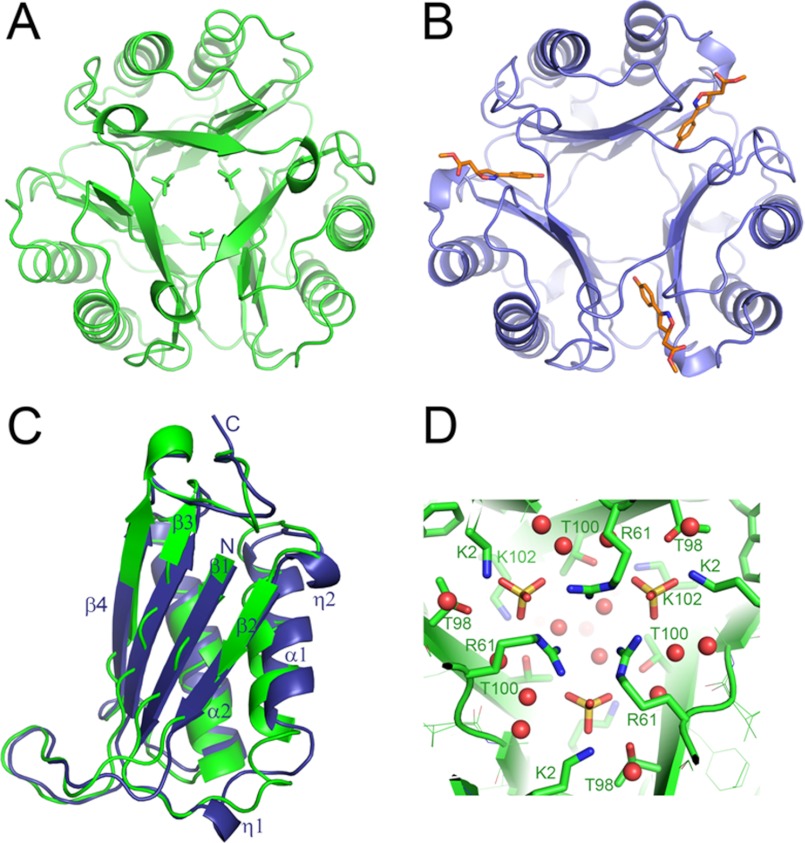
*A*, the x-ray crystal structures of *T. gondii* MIF (*green*) (Protein Data Bank code 4DH4) with the three sulfate ions bound in the pore shown in *stick* representation. *B*, human MIF (Protein Data Bank code 1LJT), a representative of mammalian MIFs in schematic representation showing the trimeric ring architecture. The ligand ISO-1 (*orange*) is shown in *stick* representation in the active site. *C*, overlay of the monomer structure of *T. gondii* MIF (*green*) and human MIF (*blue*) with secondary structure elements labeled. *D*, sulfate ions (*yellow centered tetrahedra*) and water molecules (*red spheres*). Amino acids in the central pore making contacts with the sulfate ions are labeled.

Crystals formed in space group H3(2) and x-ray diffraction data were collected at the Diamond Light Source (Table 1). The structure of TgMIF was determined to 1.82-Å resolution. Secondary structure elements are shown above the alignments in Fig. 1. TgMIF forms a crystallographic trimer, and each monomer consists of a four-stranded mixed β-sheet and two α-helices stacked against a β-sheet (Fig. 2, *A* and *B*). The topology of the TgMIF trimer (a ring with a central pore, coincident with the molecular 3-fold axis) is conserved with HsMIF as seen in superimposition images (Fig. 2*C*).

**TABLE 1:**
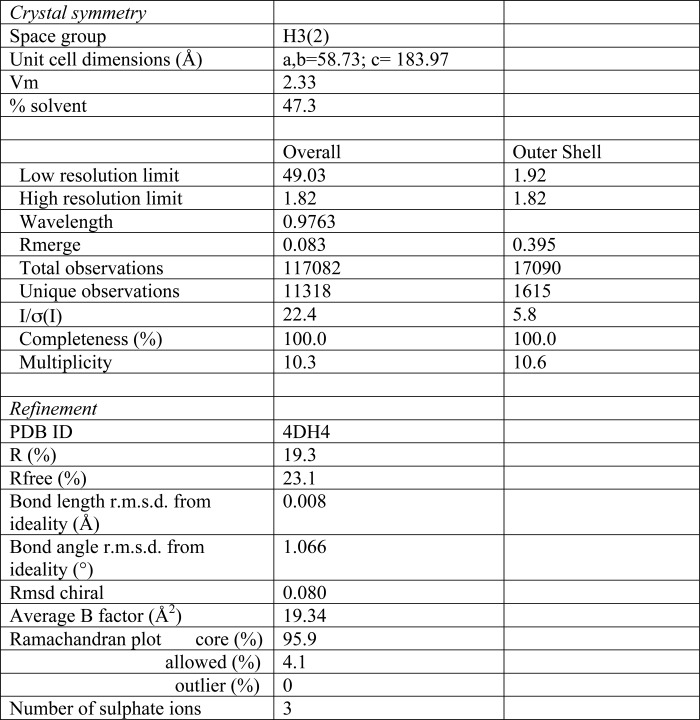
**Crystallographic data and refinement statistics** r.m.s.d., root mean square deviation.

There are interesting differences between the TgMIF and mammalian MIF structures. First, the short η1 turn, evident in mammalian MIFs (Fig. 1*A*), is absent in the TgMIF structure. Second, Pro^15^, which is conserved in mammalian MIFs and functions as a helix “breaker” between the η1 turn and α1 helix in HsMIF (19), is substituted with Glu^16^ in TgMIF. As a result, the α1 helix in TgMIF is longer than in HsMIF, extending from Pro^13^ to Leu^31^. Third, the central pore of the TgMIF trimer is narrower than that of mammalian MIFs with a minimum pore diameter of 3.5 Å at Arg^61^. Moreover, three sulfate ions were bound to positively charged residues lining the pore, notably Arg^61^ and Lys^2^, as well as a network of water molecules (Fig. 2*D*). The mammalian MIF pore is most often described as a unique solvent channel; it has been proposed that its positively charged electrostatic surface may be involved in transportation of negatively charged ions (26) or provide a binding site for negatively charged ligands such as dopachrome (26) or sialic acid-containing glycolipids important in the human monocyte response to MIF (27). However, to date, the pore has no assigned function; and therefore, it is difficult to determine the biological consequences of the differences in pore diameter in TgMIF and the role of the bound sulfate ions.

##### TgMIF Has Tautomerase Activity but No Oxidoreductase Activity

**FIGURE 3.**
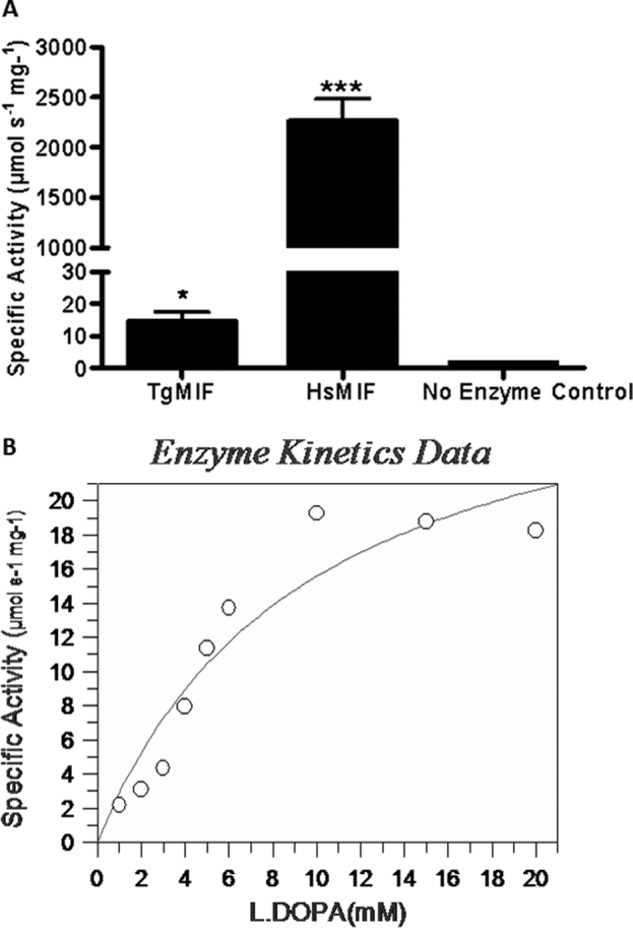
*A*, TgMIF has a tautomerase activity. Recombinant protein was expressed and purified, and TgMIF tautomerase activity was measured with 10 mm l-DOPA. Recombinant HsMIF was used as a positive control. All assays were carried out in triplicate, and data shown are means ± S.E. (*error bars*). *, *p* < 0.05; ***, *p* < 0.0001. *B*, TgMIF tautomerase kinetics were determined using recombinant protein and concentrations of l-DOPA ranging from 1 to 20 mm. All enzyme assays were carried out in triplicate.

Mammalian MIF has been shown to catalyze tautomerization reactions in which isomeric organic compounds are readily interconverted (28). We have found TgMIF tautomerase activity to be stable for 4 days following purification (data not shown). As it has been suggested that MIF may mediate its biological functions via enzyme activity, only freshly purified protein was used in further biochemical and immunological assays. In a representative run, TgMIF tautomerase activity toward substrate l-DOPA methyl ester exhibited a specific activity of 19.3 μmol/min/mg of protein, which is 104- and117-fold lower than that of mouse (2011 μmol s^−1^ mg^−1^; Ref. 19) and human (2262 μmol s^−1^ mg^−1^; this study) MIF, respectively (Fig. 3*A*). TgMIF tautomerase kinetics were fully characterized during which a *K_m_* of 9.6 mm, *V*_max_ of 30 μmol s^−1^ mg^−1^, and *K*_cat/_*K_m_* of 13.99 s^−1^ m^−1^ were measured (Fig. 3*B*). The oxidoreductase activity in TgMIF could not be detected up to 2 h after addition of protein (data not shown).

##### TgMIF Tautomerase Activity Is Not Inhibited by ISO-1

**FIGURE 4.**
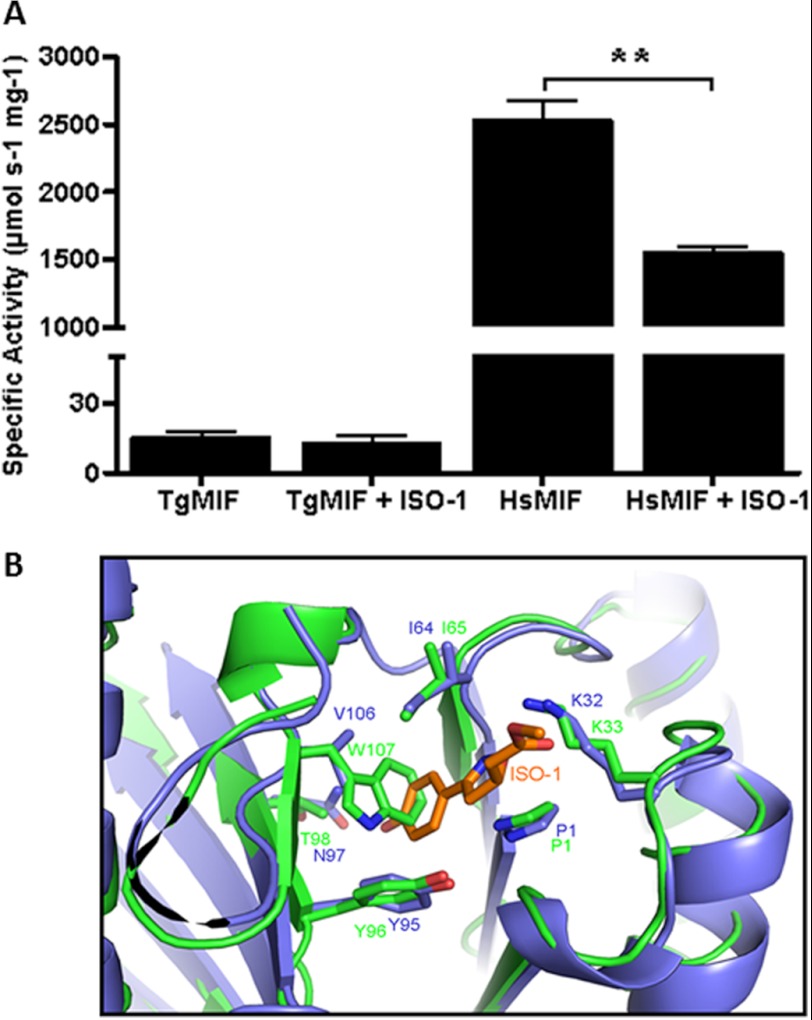
*A*, TgMIF tautomerase activity was not affected by preincubation with inhibitor ISO-1. HsMIF tautomerase activity was reduced by ∼60% following preincubation with ISO-1. Assays were carried out in triplicate, and data shown are means ± S.E. (*error bars*). **, *p* < 0.001. *B*, prediction of interactions between active site amino acids in TgMIF (*green*) and HsMIF (*blue*) with ISO-1 that prevent or accommodate ISO-1 inhibition, respectively.

TgMIF tautomerase activity was not significantly inhibited by preincubation with inhibitor ISO-1, reflecting observations in other parasite MIF homologues (18, 29). HsMIF tautomerase activity, on the other hand, was inhibited by 60% following preincubation with 10 μm ISO-1 (Fig. 4*A*).

Structural analysis reveals amino acid substitutions in the area of the tautomerase active site that could ultimately alter the catalytic activity of TgMIF. Mammalian MIF tautomerase activity involves the Pro^1^, Lys^32^, and Ile^64^ of one monomer in an interaction with Tyr^95^ and Asn^97^ (corresponding to Pro^1^, Lys^34^, Ile^65^, Tyr^96^, and Thr^98^ in the TgMIF sequence) of the adjacent monomer (30).

Most amino acids of the active site are conserved in addition to Tyr^37^ and Phe^114^, which surround the active site. However, replacement of Asn^97^ for Thr^98^ in the TgMIF tautomerase active site is of importance as this substitution prevents the formation of a hydrogen bond between MIF and the substrate molecule, l-DOPA methyl ester (19). An identical substitution has been reported previously in the *Ancylostoma ceylanicum* MIF homologue with the suggestion that this substitution may account for a weakened tautomerase activity (29). Further substitutions around the TgMIF tautomerase active site are observed whereby Val^106^ is replaced by Trp^107^, resulting in possible steric hindrance that prevents the successful binding of ISO-1 to the active site (Fig. 4*B*).

##### Endotoxin-free TgMIF Drives IL-8 Production from Human PBMCs and Induces ERK MAPK Activation in Bone Marrow-derived Macrophages

**FIGURE 5.**
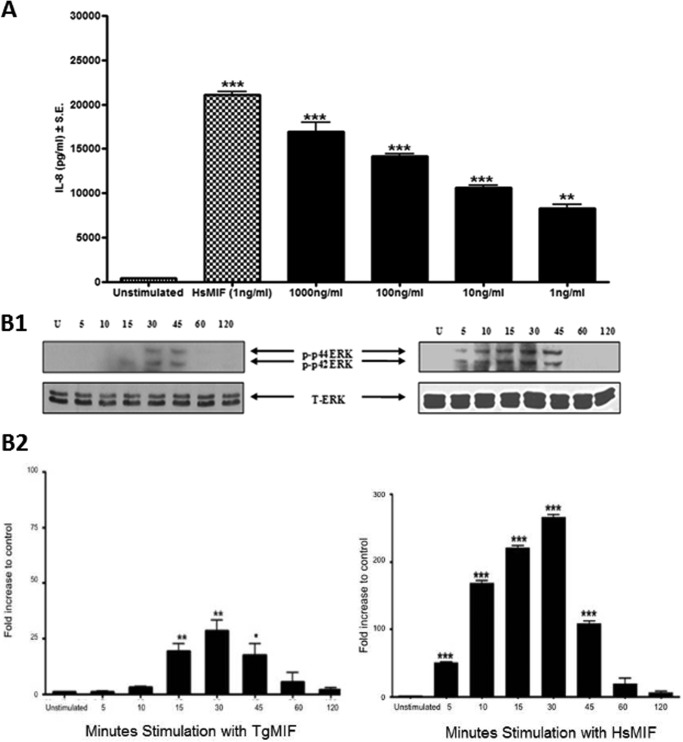
*A*, TgMIF induces IL-8 production from human peripheral blood mononuclear cells in a dose-dependent manner. Data shown are from one individual, although the experiment was repeated using samples from a further two individuals whereby the same trend in IL-8 production was observed. Data shown are means of replicates for one assay ±S.E. (*error bars*). *B*, TgMIF activates ERK MAPK pathways in bone marrow-derived macrophages in a time-dependent manner (*panel 1*). Activation peaks at 30 min poststimulation when stimulated by both TgMIF and HsMIF (*panel 2*). “*U*” indicates cells that were not stimulated throughout the experiment. “*p*” indicates phosphorylated ERK isoforms. “*T*” indicates total phosphorylated ERK. All experiments were carried out in triplicate, and data shown are representative of means ± S.E. (*error bars*). *, *p* < 0.05; **, *p* < 0.001; ***, *p* < 0.0001.

LPS in recombinant TgMIF was less than 0.008 ng/mg of protein (data not shown). TgMIF can drive the production of IL-8 in human PBMCs in a dose-dependent manner and is biologically active at 1 ng/ml. However, whereas 1 ng/ml HsMIF induced 22.5 ng/ml (S.E., ±0.545 ng/ml) IL-8 production from PBMCs, TgMIF induced 8.0 ng/ml IL-8 (S.E., ±0.721 ng/ml). Consequently, TgMIF was ∼40% as effective at stimulating IL-8 as equivalent levels of HsMIF from PBMCs (Fig. 5*A*).

An increase in ERK MAPK activation was observed in murine bone marrow-derived macrophages stimulated with TgMIF at a final concentration of 100 ng/ml. The increase in ERK MAPK activation was represented by an increase in phosphorylation of p42/p44 ERK up to 30 min poststimulation, and thereafter phosphorylation decreased. After 30-min stimulation with TgMIF, there was a ∼30-fold increase in ERK MAPK activation compared with unstimulated control (Fig. 5*B*). The same pattern of activation was observed following stimulation of macrophages with 1 ng/ml HsMIF whereby phosphorylation peaked after 30 min of stimulation when phosphorylated ERK increased ∼270-fold compared with unstimulated cells (Fig. 5*B*).

## DISCUSSION

Since its discovery in 1966 (31, 32), MIF has emerged as an important and often critical mediator of the immune response (33–35). MIF has been described as a diverse cytokine with potent proinflammatory properties, which are potentially mediated via its unique enzyme activities (36). However, there are conflicting reports as to whether the tautomerase activity can facilitate MIF biological activities (16, 37, 38). In recent years, there have been studies describing homologues of MIF found in many parasitic species including some helminths and protozoa including *Plasmodium*, *Leishmania*, and *Brugia* species (17–19, 39). We report herein the characterization of the MIF homologue in the *T. gondii* RH strain that has 26% amino acid sequence identity to mammalian MIFs described herein. Production of recombinant TgMIF has facilitated structure determination as well as characterization of its biochemical properties and immunomodulatory capabilities.

MIF homologues from all species examined to date have been demonstrated to catalyze the tautomerization of the substrate l-DOPA methyl ester. This has been shown to be dependent on the N-terminal proline, which is also conserved in the TgMIF (40). Consistent with this, TgMIF exhibits measurable tautomerase activity; albeit this was significantly less than that observed for HsMIF in identical assay conditions. TgMIF tautomerase activity is of the same order as observed previously for *Leishmania major* MIF under the same assay conditions (19). *Trichinella spiralis* MIF has been reported to have tautomerase activity similar to those of TgMIF and LmMIF (39). However, extrapolation between studies should be done with caution because of differences in experimental protocol.

Of note, TgMIF lacks an active oxidoreductase activity, which is similar to other parasite MIF homologues (19). This is likely to be caused by the absence of a second cysteine residue in the C*XX*C motif at position 60 that is necessary for optimum activity (41).

Analysis of the TgMIF structure reveals key differences compared with mammalian MIFs (including human, cat, and murine) around the tautomerase active site. These differences might account for poor tautomerase activity and the subsequent inability of the competitive inhibitor ISO-1 to bind to this site. There are five key amino acids in the catalytic site associated with tautomerase activity. However, reduced catalytic ability in the TgMIF is predicted to be attributed to a substitution of Asn^97^ for a Thr, which along with the steric hindrance caused by the replacement of Val^106^ with Tyr prevents easy access of the substrate/inhibitor to the active site. Notably, the MIF homologue of *A. ceylanicum* is also unaffected by ISO-1. The discriminative nature in which ISO-1 selectively binds to HsMIF and not TgMIF (or other parasite MIFs) highlights significant differences in the tautomerase active site that could potentially be exploited in the development of new antiparasite inhibitors (18, 29).

MIF has been associated with a diverse variety of biological processes including its ability to counteract the immunosuppressive properties of glucocorticoids (12) and to induce angiogenesis (42). However, a recent study (13) has highlighted the inconsistency in reporting endotoxin removal procedures and the presence of residual endotoxin levels present in protein batches in MIF studies. Subsequently, the production of LPS-free recombinant TgMIF was essential to provide reliable and robust data; and therefore, we ensured that all protein used in biological assays had negligible levels of residual LPS.

The initial description of MIF was as a soluble mediator secreted by the T cells of rodents that inhibited the random migration of monocytes and directed them to the site of inflammation (31, 32). More recent studies have demonstrated that MIF activates ERK pathways in both mice and humans to induce production of the chemokine IL-8 (43, 44). Expression of the murine homologue of IL-8, MIP-2, is similarly dependent on activation of ERK MAPK (45), and significantly in the present study, we have demonstrated that ERK signaling pathways are promoted by TgMIF as well as HsMIF. IL-8 as well as its murine homologue MIP-2 is secreted by many cells such as endothelial and epithelial cells in response to challenge by foreign antigen and functions mainly as a chemoattractant for cells of the immune response including macrophages but primarily neutrophils (46). HsMIF has been shown previously to induce IL-8 production from THP-1 cells and human PBMCs (13). In this study, we also confirmed the ability of recombinant endotoxin-free TgMIF to induce IL-8 production from human PBMCs in a dose-dependent manner with TgMIF having activity roughly comparable to that of HsMIF (40%). In addition, IL-8 production has been observed in studies of other MIF homologues; *e.g.* both *Brugia malayi* MIF homologues have been shown to induce IL-8 production from human monocytes (20). Furthermore, IL-8 production from fibroblasts and HeLa cells has been reported from cells infected with *T. gondii* tachyzoites (47). Significantly, it has been observed in the acute stages of *T. gondii* infection that there is a rapid recruitment of neutrophils to the site of infection (48), and this has been demonstrated to be essential for resolution of infection. Paradoxically, the early recruitment of neutrophils to the site of infection may also aid the spread of infection as *T. gondii* has been shown to trigger neutrophil synthesis of CC chemokine ligand (CCL) 3, CCL4, CCL5, and CCL20 (49). These are strongly chemotactic for immature dendritic cells, subpopulations of which have been described in recent studies as Trojan horses to facilitate *T. gondii* dissemination (50, 51). Consequently, TgMIF may not only serve to limit the severity of infection, which would be beneficial for host survival, but also to facilitate parasite dissemination to host tissue, which would promote the potential successful completion of the parasite life cycle.

Overall, our studies demonstrate the presence of a MIF homologue in the *T. gondii* genome that is enzymatically and immunologically active *in vitro*. We also hypothesize a role for this parasite protein in modulating host immune responses to provide a favorable environment suitable for long term parasite residence, potentially exerting these effects by interactions with the human MIF receptor, CD74.

## Footnotes

2 The abbreviations used are:

MIF: migration inhibitory factor
TgMIF: *T. gondii* MIF
PBMC: human peripheral blood mononuclear cell
DOPA: 3,4-dihydroxyphenylalanine
HsMIF: human MIF
ISO-1: (*S*,*R*)-3-(4-hydroxyphenyl)-4,5-dihydro-5-isoxazole acetic acid
LmMIF: *L. major* MIF
CCL: CC chemokine ligand.
